# A two-year survey of the oseltamivir-resistant influenza A(H1N1) virus in Yamagata, Japan and the clinical effectiveness of oseltamivir and zanamivir

**DOI:** 10.1186/1743-422X-7-53

**Published:** 2010-03-05

**Authors:** Yoko Matsuzaki, Katsumi Mizuta, Yoko Aoki, Asuka Suto, Chieko Abiko, Kanako Sanjoh, Kanetsu Sugawara, Emi Takashita, Tsutomu Itagaki, Yuriko Katsushima, Makoto Ujike, Masatsugu Obuchi, Takato Odagiri, Masato Tashiro

**Affiliations:** 1 Course of Clinical Nursing, Yamagata University Faculty of Medicine, Yamagata 990-9585, Japan; 2 Yamagata Prefectural Institute of Public Health, Yamagata 990-0031, Japan; 3 Sanjoh Clinic, Yamagata 996-0084, Japan; 4 Department of Infectious Diseases, Yamagata University Faculty of Medicine, Yamagata 990-9585, Japan; 5 Influenza Virus Research Center, National Institute of Infectious Diseases, Tokyo 208-0011, Japan; 6 Yamanobe Pediatric Clinic, Yamagata 990-0301, Japan; 7 Katsushima Pediatric Clinic, Yamagata 990-2461, Japan

## Abstract

**Background:**

Oseltamivir is the preferred antiviral drug for influenza, but oseltamivir-resistant A(H1N1) viruses have circulated worldwide since the 2007-2008 influenza season. We aimed to determine the rate of oseltamivir resistance among A(H1N1) isolates from Yamagata, Japan, to compare the virological characteristics between isolates from the 2007-2008 and 2008-2009 seasons, and to evaluate the clinical effectiveness of oseltamivir.

**Results:**

Oseltamivir resistance, determined by detecting the H275Y mutation in the neuraminidase (NA) gene, was observed in 2.5% (2 of 79) and 100% (77 of 77) of isolates from the 2007-2008 and 2008-2009 seasons, respectively. Antigenic analysis suggested that antigenically different variants of A(H1N1) viruses circulated in the 2008-2009 season. Growth testing demonstrated that the ability of the 2008-2009 isolates to replicate in MDCK cells was similar to those of the oseltamivir-susceptible isolates from the 2007-2008 season. A phylogenetic analysis revealed that two oseltamivir-resistant viruses isolated in the 2007-2008 season were closely related to other oseltamivir-susceptible viruses in Yamagata but were different from oseltamivir-resistant viruses isolated in Europe and North America in the 2007-2008 season. The oseltamivir-resistant viruses isolated in Japan in the 2008-2009 season were phylogenetically similar to oseltamivir-resistant isolates from Europe and North America during the 2007-2008 season. Furthermore, the median duration of fever after the start of oseltamivir treatment was significantly longer in oseltamivir-resistant cases (2 days; range 1-6 days) than in oseltamivir-susceptible cases (1.5 days: range 1-2 days) (*P *= 0.0356).

**Conclusion:**

Oseltamivir-resistant A(H1N1) isolates from Yamagata in the 2007-2008 season might have acquired resistance through the use of oseltamivir, and the 2008-2009 oseltamivir-resistant isolates might have been introduced into Japan and circulated throughout the country. Influenza surveillance to monitor oseltamivir-resistance would aid clinicians in determining an effective antiviral treatment strategy.

## Background

During the 2007-2008 season, increased levels of resistance to oseltamivir among influenza A (H1N1) viruses were reported in Europe and North America [1-6], and oseltamivir-resistant viruses were also detected in the southern hemisphere [7,8]. The frequency of oseltamivir-resistance in A(H1N1) isolates was highest (67%) in Norway [9]. During the same season in Japan, it is estimated that up to 2.6% of all influenza A(H1N1) isolates were resistant to oseltamivir [10]. It was reported that some of the resistant viruses found in Japan during the 2007-2008 season were not phylogenetically related to those found in Europe and that these resistant isolates from Japan emerged independently in Japan [11,12]. Further, during the 2008-2009 season, the A(H1N1) virus was prominent in influenza outbreaks in Japan, and national surveillance showed that 99.6% of A(H1N1) isolates had the histidine-to-tyrosine substitution at residue 275 (H275Y) of the neuraminidase (NA) gene; this mutation is associated with oseltamivir resistance [13]. Oseltamivir is widely used in clinical settings in Japan. Therefore, an increase in oseltamivir-resistant influenza viruses is an important problem that is likely to influence the treatment strategy for influenza virus infections.

The purposes of this study were to investigate the percentage of A(H1N1) isolates from Yamagata Prefecture during the 2007-2008 and 2008-2009 seasons that had the H275Y mutation in the NA gene and to compare the virological characteristics between the A(H1N1) viruses isolated in those seasons. Additionally, we evaluated the clinical effectiveness of oseltamivir and zanamivir against oseltamivir-resistant A(H1N1) virus infections.

## Results

### The percentage of influenza A(H1N1) virus isolates with the H275Y mutation

A total of 156 isolates from the Yamagata prefecture obtained between December 2007 and March 2008 (2007-2008 isolates) and between December 2008 and March 2009 (2008-2009 isolates) were sequenced for the identification of the H275Y mutation in the NA gene. The sequencing results demonstrated that 2.5% of the 2007-2008 isolates and 100% of 2008-2009 isolates had the H275Y mutation associated with oseltamivir resistance (Table 1).

**Table 1:** Influenza A(H1N1) virus resistance to oseltamivir in Yamagata, Japan

Season	Total tested	Number (%) of oseltamivir-resistant isolates neuraminidase H275Y mutation
Dec 2007-Mar 2008	79	2 (2.5)
Dec 2008-Mar 2009	77	77 (100)

### The NA inhibition assay against oseltamivir and zanamivir

Seven isolates were tested for susceptibility to the NA inhibitors oseltamivir and zanamivir. Two 2007-2008 isolates and two 2008-2009 isolates with the H275Y mutation showed 234- to 1,968-fold reductions in susceptibility to oseltamivir when compared with three 2007-2008 isolates without the H275Y mutation (Table 2). However, the H275Y mutation had no impact on the susceptibility to zanamivir.

**Table 2:** Inhibition of the enzyme activity of the A(H1N1) isolates in Yamagata in the NA inhibition assay

Viruses	Amino Acid at position 275 in the NA gene	IC_50_ values in the NA inhibition assay (nM)	
		
		Oseltamivir	Zanamivir
2007-2008 isolates			
A/Yamagata/1/2008	Histidine	0.08	0.52
A/Yamagata/66/2008	Tyrosine	50.16	0.45
A/Yamagata/67/2008	Histidine	0.03	0.22
A/Yamagata/68/2008	Tyrosine	39.71	0.55
A/Yamagata/69/2008	Histidine	0.17	0.78
			
2008-2009 isolates			
A/Yamagata/126/2008	Tyrosine	59.04	0.50
A/Yamagata/128/2008	Tyrosine	47.59	0.40

### Antigenic analysis

The antigenic analysis was performed by hemagglutination inhibition (HI) tests for reactivity with post-infection ferret antisera against two A(H1N1) vaccine strains (A/Solomon Islands/3/2006 [2007-2008 vaccine strain] and A/Brisbane/59/2007 [2008-2009 vaccine strain]) (Table 3). A/Yamagata/66/2008 and A/Yamagata/68/2008, the oseltamivir-resistant 2007-2008 isolates, were antigenically similar to the other oseltamivir-susceptible 2007-2008 isolates. However, oseltamivir-resistant 2008-2009 isolates except A/Yamagata/45/2009 showed a fourfold decrease in the HI titer compared with oseltamivir-resistant 2007-2008 isolates, indicating that antigenically different variants of oseltamivir-resistant A(H1N1) viruses circulated in Yamagata in the 2008-2009 season.

**Table 3:** Antigenic analysis of the A(H1N1) isolates in Yamagata by the HI test

	HI titer of post-infection ferret sera to:	
	
Viruses	A/Solomon Islands/3/2006	A/Brisbane/59/2007
A(H1N1) vaccine strains		
A/Solomon Islands/3/2006	160	160
A/Brisbane/59/2007	160	160
		
2007-2008 isolates		
A/Yamagata/1/2008	80	160
A/Yamagata/66/2008	160	160
A/Yamagata/67/2008	160	320
A/Yamagata/68/2008	160	160
A/Yamagata/69/2008	160	160
		
2008-2009 isolates		
A/Yamagata/125/2008	20	10
A/Yamagata/126/2008	40	40
A/Yamagata/128/2008	40	40
A/Yamagata/45/2009	20	160
A/Yamagata/80/2009	20	40

### Virus growth in MDCK cells

**Figure 1:**
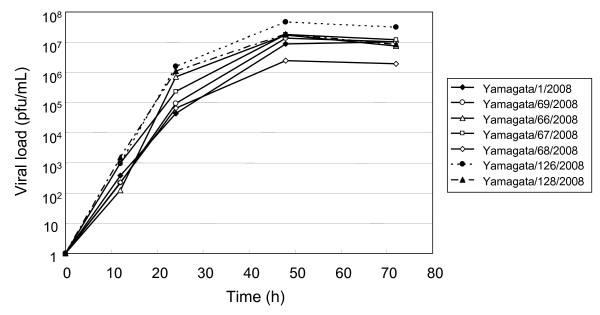
**Growth of oseltamivir-susceptible or -resistant A(H1N1) viruses from the 2007-2008 season and 2008-2009 seasons in Yamagata**. A/Yamagata/66/2008 and A/Yamagata/68/2008 from the 2007-2008 season and A/Yamagata/126/2008 and A/Yamagata/128/2008 from the 2008-2009 season were all oseltamivir-resistant viruses. Viruses were grown in MDCK cells, and the supernatants were harvested at the indicated time points and titrated by plaque assays. Growth tests were performed in duplicate experiments, and the mean virus titers are shown in Figure.

We compared the growth characteristics of oseltamivir-resistant 2008-2009 isolates with those of oseltamivir-susceptible and -resistant 2007-2008 isolates. As shown in Figure 1, the virus titers of A/Yamagata/126/2008 and A/Yamagata/128/2008, which were oseltamivir-resistant 2008-2009 isolates, increased, as did those of the oseltamivir-susceptible isolates from the 2007-2008 season. The two 2008-2009 isolates showed rapid growth and A/Yamagata/126/2008 reached a more than ten-fold higher final virus titer than did A/Yamagata/68/2008, an oseltamivir-resistant virus from the 2007-2008 season.

### Phylogenetic analysis of the HA and NA genes

**Figure 2:**
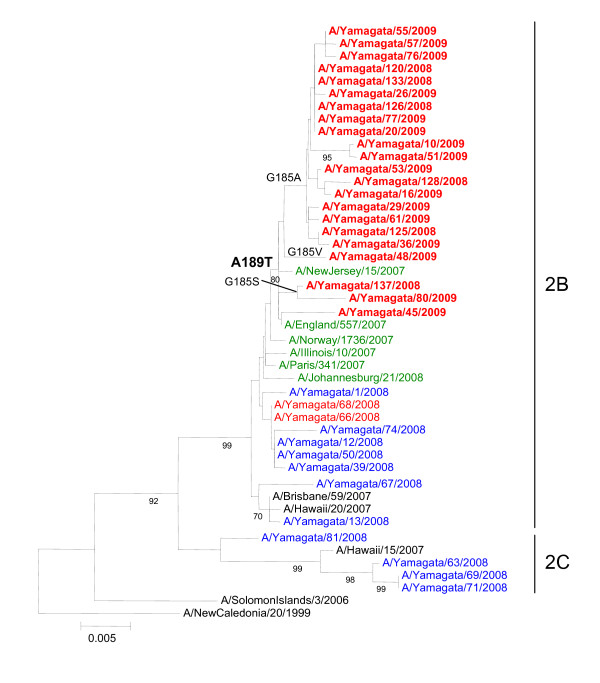
**Phylogenetic trees for the HA gene of A(H1N1) viruses**. The region from nucleotide 33 to 1102 (1070 nucleotides) for the HA gene were used for the analysis. The numbers below the branches are the bootstrap probabilities (percentages), showing only values greater than 70%. Viruses with a H275Y mutation in the NA gene isolated in Yamagata are shown in red, and those isolated in Europe, the USA, and South Africa are shown in green. Viruses with H275 strains in Yamagata are shown in blue. Viruses from the 2008-2009 season are indicated in boldface.

**Figure 3:**
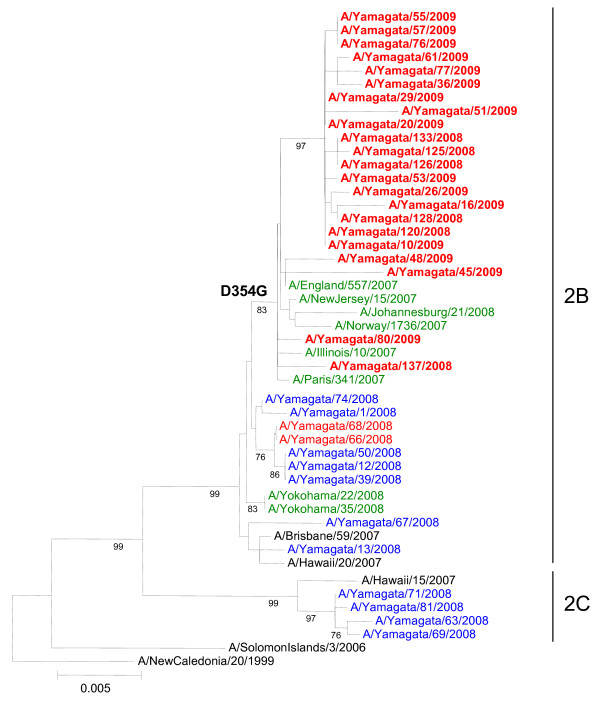
**Phylogenetic trees for the NA gene of A(H1N1) viruses**. The region from nucleotide 21 to 1430 (1410 nucleotides) for the NA gene were used for the analysis. The numbers below the branches are the bootstrap probabilities (percentages), showing only values greater than 70%. Viruses with a H275Y mutation in the NA gene isolated in Yamagata are shown in red, and those isolated in Europe, the USA, South Africa, and Japan (except Yamagata) are shown in green. Viruses with H275 strains in Yamagata are shown in blue. Viruses from the 2008-2009 season are indicated in boldface.

Of the 156 A(H1N1) isolates, 13 isolates from the 2007-2008 season and 22 isolates from the 2008-2009 season were used for the phylogenetic analysis. All of the 2007-2008 isolates belonged to two distinct lineages (2B and 2C) in the HA (Figure 2) and NA (Figure 3) gene trees. Two oseltamivir-resistant 2007-2008 isolates, A/Yamagata/66/2008 and A/Yamagata/68/2008, were isolated three days apart from two respective students from the same elementary school who had not been treated with oseltamivir prior to the specimen collection. The nucleotide sequences of both the HA and NA genes of these two viruses were identical, and these viruses showed close genetic similarity to the oseltamivir-susceptible 2007-2008 isolates belonging to 2B. The oseltamivir-resistant A(H1N1) viruses that emerged in Europe in late 2007 are characterized by D354G amino acid substitutions in the NA protein [2]. However, oseltamivir-resistant 2007-2008 isolates from Yamagata did not have the D354G mutation in the NA gene.

On both the HA and NA phylogenetic trees, all oseltamivir-resistant 2008-2009 isolates from Yamagata belonged to the 2B lineage and were more closely related to the oseltamivir-resistant viruses isolated in Europe and North America in the 2007-2008 season than to the oseltamivir-resistant 2007-2008 isolates from Yamagata. All 2008-2009 isolates have the D354G mutation in the NA gene and the A189T mutation in the HA gene. Except for A/Yamagata/45/2008, all 2008-2009 isolates from Yamagata have an amino acid substitution at residue 185 (G185A, G185V, or G185S) of the HA protein. This substitution might influence the decrease in the reactivity with antiserum against A/Brisbane/59/2007.

### Clinical effectiveness of oseltamivir and zanamivir

We investigated the clinical effectiveness of anti-influenza drugs by comparing the symptoms of children with oseltamivir-susceptible influenza A(H1N1) virus infections during the 2007-2008 season to those of children with oseltamivir-resistant influenza A(H1N1) virus infections during the 2008-2009 season (Table 4). We found no significant differences in the age, maximum temperature, or total febrile period of oseltamivir-treated children between cases of the oseltamivir-susceptible and oseltamivir-resistant influenza A(H1N1) virus infections. However, for the children treated with oseltamivir, the duration of fever after the start of treatment was significantly longer in children with oseltamivir-resistant influenza A(H1N1) virus infections during the 2008-2009 season than in children with oseltamivir-susceptible influenza A(H1N1) virus infections during the 2007-2008 season, though no significant differences were observed among the zanamivir-treated children.

**Table 4:** Comparison of the effectiveness of oseltamivir and zanamivir against the oseltamivir-susceptible and oseltamivir-resistant influenza A(H1N1) infections

	Oseltamivir-treated cases			Zanamivir-treated cases		
				
	2007-2008 Oseltamivir-susceptible (n = 8)	2008-2009 Oseltamivir-resistant (n = 25)	P-value	2007-2008 Oseltamivir-susceptible (n = 4)	2008-2009 Oseltamivir-resistant (n = 6)	P-value
Age, years; median (range)	6 (1-9)	5 (2-9)	.8155	8 (7-11)	11 (9-12)	.0506
Maximum body temperature, °C; median (range)	39.3 (38.9-39.8)	39.1 (38.2-40.6)	.3874	39.2 (38.6-39.6)	39.0 (38.2-39.6)	.5906
Total febrile period, days; median (range)	3.5 (2-5)	4 (2-7)	.0503	3 (2-4)	3 (3-4)	.4642
Duration of fever after the start of therapy, days; median (range)	1.5 (1-2)	2 (1-6)	.0356	1 (1-2)	1 (1-3)	.6926

## Discussion

Oseltamivir is widely used for patients with influenza A and B infections in clinical settings in Japan. Therefore, oseltamivir-resistant viruses induced by oseltamivir treatment emerge in Japan more readily than in other countries where oseltamivir is not so widely used. It has been documented that the infectivity and replicative ability of neuraminidase inhibitor-resistant viruses are compromised [14,15]; therefore, until recently it was thought that oseltamivir-resistant influenza A(H1N1) viruses were unlikely to circulate among humans.

Antigenic and phylogenetic analyses in this study revealed that two oseltamivir-resistant influenza A(H1N1) virus strains isolated in the 2007-2008 season (A/Yamagata/66/2008 and A/Yamagata/68/2008) were closely related to other oseltamivir-susceptible A(H1N1) viruses isolated in Yamagata but were different from the resistant viruses found in Europe. It appears that oseltamivir-resistant viruses found during the 2007-2008 season in Japan emerged independently among persons treated with oseltamivir and were isolated in various communities, as previously described [11,12]. Two oseltamivir-resistant viruses in the present study have HA and NA genes sequences that are 100% identical between the two isolates; these viruses were isolated on February 29 and March 3 from two different children from the same elementary school who had not been treated with oseltamivir. In this school, an outbreak of influenza occurred on February 29, 2008, and 28 (22%) of 129 students showed influenza-like symptoms between February 29 and March 17, 2008. Although only two samples were collected from which oseltamivir-resistant viruses were isolated, there is a possibility that the transmission of an oseltamivir-resistant virus had occurred in this school. It was also reported that an outbreak of an oseltamivir-resistant A(H1N1) virus occurred in an elementary school in Yokohama City in Japan during the same season (A/Yokohama/22/2008 and A/Yokohama/35/2008 in the NA gene tree of Figure 3) [12]. Thus, it is likely that oseltamivir-resistant viruses posses the ability to be transmitted among humans. However, because the level of oseltamivir resistance remained at 2.5% in Yamagata (2.6% in Japan), it is apparent that the oseltamivir-resistant viruses did not spread among humans as easily as the oseltamivir-susceptible viruses.

The oseltamivir-resistant viruses isolated during the 2008-2009 season in Yamagata were antigenically and phylogenetically different from those isolated during the 2007-2008 season in Yamagata but were phylogenetically similar to viruses isolated in Europe and North America during the 2007-2008 season. Thus, it seems that oseltamivir-resistant viruses were imported into Japan, where they spread across the country during the 2008-2009 season. Some reports have suggested that the emergence of oseltamivir-resistant viruses in the 2007-2008 season in Europe was not related to the use of oseltamivir [16,17], and that natural genetic variations may have resulted in the change in sensitivity to oseltamivir [18]. It is likely that influenza A(H1N1) viruses having an epidemiological advantage over previous viruses emerged with natural resistance to oseltamivir and spread throughout the world. Two representative viruses isolated during the 2008-2009 season in Yamagata (A/Yamagata/126/2008 and A/Yamagata/128/2008) showed the same level of growth in MDCK cells as the oseltamivir-susceptible 2007-2008 isolates. The maintenance of replicative ability and the acquirement of antigenic differences are vital for viruses to continue transmission among humans. Hereafter, A(H1N1) viruses might continue to circulate with the H275Y mutation in the NA gene preserved. The pandemic H1N1 viruses emerged in 2009 were oseltamivir-susceptible without H275Y mutation in the NA gene. Cocirculation of oseltamivir-resistant seasonal A(H1N1) viruses and the novel H1N1 pandemic viruses may give rise to the potential risk of genetic reassortment acquiring the H275Y resistance mutation in the NA gene.

There have been few reports concerning the clinical effectiveness of oseltamivir against oseltamivir-resistant influenza viruses. We found that the duration of fever after the start of oseltamivir treatment was significantly longer in children with oseltamivir-resistant influenza A(H1N1) virus infections than in children with oseltamivir-susceptible A(H1N1) virus infections. This result suggests that oseltamivir-resistant A(H1N1) viruses circulating during the 2008-2009 season were resistant to oseltamivir not only *in vitro *but also *in vivo*. Zanamivir, however, reduced the duration of fever in oseltamivir-resistant cases. In Japan, zanamivir is used as well as oseltamivir, especially for the treatment of patients older than five years. Thus, in cases of oseltamivir-resistant influenza virus infection, zanamivir would be a more appropriate treatment than oseltamivir.

## Conclusions

Oseltamivir resistance was observed in 2.5% and 100% of A(H1N1) isolates from Yamagata from the 2007-2008 and 2008-2009 seasons, respectively. Oseltamivir-resistant isolates from Yamagata in the 2007-2008 season might have acquired resistance through the use of oseltamivir. In contrast, the 2008-2009 oseltamivir-resistant isolates from Yamagata were similar to viruses isolated in Europe and North America; therefore, they might have been introduced into Japan and circulated throughout the country. It is certain that influenza surveillance to monitor oseltamivir resistance will benefit clinicians in determining an effective antiviral treatment strategy. Timely monitoring and reporting of antiviral drug-resistance is important at the global, national, and the local community levels.

## Methods

### Specimen collection and virus isolation

Nasopharyngeal swab specimens from patients with acute respiratory infection were collected at pediatric clinics collaborating with the local health authorities in Yamagata Prefecture for the surveillance of viral diseases in Japan. Specimens were transported to the Yamagata Prefectural Institute of Public Health and were grown in a virus culture as previously described [19]. A total of 79 A(H1N1) viruses isolated from 690 specimens between December 2007 and March 2008 and a total of 77 A(H1N1) viruses isolated from 831 specimens between December 2008 and March 2009 were used in this study. Of the 156 isolates, 84 (53.8%) were isolated from children under 6 years of age, 55 (35.3%) were from children aged between 6 and 10 years, 15 (9.6%) were from children aged between 11 and 15 years, and 2 (1.3%) were from patients >15 years.

### NA inhibition assay

A chemiluminescent NA inhibition assay was performed using a commercially available kit, NA-Star (Applied Biosystems, Foster City, CA), according to the manufacturer's protocol. The NA inhibitors (oseltamivir and zanamivir) susceptibility of influenza virus isolates was expressed as the concentration of NA inhibitor needed to inhibit the NA enzyme activity by 50% (IC_50_). Oseltamivir carboxylate, the active form of the prodrug oseltamivir phosphate, was provided by F. Hoffmann-La Roche Ltd, Switzerland, and zanamivir was provided by GlaxoSmithKline Research and Development Ltd., United Kingdom.

### Hemagglutination inhibition test

The hemagglutination inhibition (HI) test was performed in microtiter plates with 1% guinea pig erythrocytes and post-infection ferret antisera against A/Solomon Islands/3/2006 [2007-2008 A(H1N1) vaccine strain] and A/Brisbane/59/2007 [2008-2009 A(H1N1) vaccine strain] (Denka Seiken Co., Ltd., Tokyo, Japan), as described previously [20]. Sera were treated with receptor-destroying enzyme before use. For the vaccine antigen, purified HA protein (Denka Seiken Co., Ltd) was diluted in a hemagglutination titer of eight, while MDCK culture fluid was also diluted in hemagglutination titer of eight and used as the isolate antigen.

### Growth of H1N1 isolates in culture

MDCK cells were infected with an A (H1N1) isolate at an MOI of 0.001 and incubated for 72 hours at 37°C in the presence of 2 μg/ml trypsin. At 12 h, 24 h, 48 h, and 72 h post-infection, the supernatants were harvested and virus titers were determined by a plaque assay on MDCK cells. The growth test was performed in duplicate experiments, and the mean virus titer was calculated.

### Sequence analyses

The extraction of viral RNA and cDNA synthesis using random primers were performed as described previously [21,22]. The cDNA was then used as the template for the amplification of the HA and NA genes by PCR. The HA gene was amplified using primers 5'-AGCAAAAGCAGGGGAAAATAA-3' and 5'-AACCATCTACCATTCCAGTC-3', and the NA gene was amplified using primers 5'-AGCAAAAGCAGGAGTTTAAAATGA-3' and 5'-GTAGAAACAAGGAGTTTTTTCAAC-3'. The PCR products were purified with a QIAquick PCR purification kit (QIAGEN, Hilden, Germany) and then sequenced using a Big Dye Terminator V1.1 cycle sequencing kit (Applied Biosystems) on an Applied Biosystems 3130 automatic sequencer. The nucleotide sequences determined in this study were assigned the accession numbers as follows at GenBank: AB521053-AB521112 and AB539701-AB539710. Published A(H1N1) virus sequences were obtained from GenBank (Accession numbers AB465320, AB465322, CY030230, CY030233, CY033622, CY033624, EU124136, EU124137, EU516057, EU516085, EU516083, EU516116, EU516118, EU516200, EU516257, EU516148, EU551811, EU551832, EU914903, EU914910, FJ403550, FJ445031, FJ445089, FJ654304). The sequence data were analyzed with the CLUSTAL W version 1.83 software, and a phylogenetic tree was constructed via the neighbor-joining method using the same software.

### Clinical information

We used the clinical information of children who visited the Sanjoh Clinic and from whom A(H1N1) influenza virus was isolated. Data were obtained retrospectively from their medical records. To compare the data between oseltamivir-resistant and oseltamivir-susceptible A(H1N1) virus infections, we used the Mann-Whitney U test. A P value of < 0.05 was regarded as statistically significant.

## Abbreviations

HA: hemagglutinin; HI: hemagglutination inhibition; MDCK: Madin Darby canine kidney; MOI: multiplicity of infection; NA: neuraminidase; PCR: polymerase chain reaction.

## Competing interests

The authors declare that they have no competing interests.

## Authors' contributions

YM was responsible for the research design, antigenic and sequence analysis, and writing of this manuscript. KM, YA, AS, and CA performed the cell culture experiments, RT-PCR, and sequencing of influenza A isolates. KS (Sanjoh), TI, and YK collected specimens from patients and clinical information. KS (Sugawara) and ET performed the growth test of viruses in culture. MU, MO, TO, and MT performed the NA inhibition assay and sequencing of viruses. KM, MO and MT participated in the study design and helped to draft the manuscript. All authors read and approved the final manuscript.
